# Prevalence, Intensity, and Correlates of Schistosomiasis and Soil-Transmitted Helminth Infections after Five Rounds of Preventive Chemotherapy among School Children in Southern Ethiopia

**DOI:** 10.3390/pathogens9110920

**Published:** 2020-11-06

**Authors:** Tigist Dires Gebreyesus, Tafesse Tadele, Kalkidan Mekete, Abbie Barry, Habtamu Gashaw, Workagegnehu Degefe, Birkneh Tilahun Tadesse, Heran Gerba, Parthasarathi Gurumurthy, Eyasu Makonnen, Eleni Aklillu

**Affiliations:** 1 Division of Clinical Pharmacology, Department of Laboratory Medicine, Karolinska Institutet, Karolinska University Hospital Huddinge,14186 Stockholm, Sweden; tigistdires@gmail.com (T.D.G.); abbie.barry@ki.se (A.B.); birknehtilahun@gmail.com (B.T.T.); 2 Ethiopian Food and Drug Authority, Addis Ababa P.O. Box 5681, Ethiopia; habtamunps@gmail.com (H.G.); pharwork1984@gmail.com (W.D.); hgerba@efda.gov.et (H.G.); 3 College of Medicine and Health Sciences, Hawassa University, Hawassa P.O. Box 1560, Ethiopia; tafetade@yahoo.com; 4 Ethiopian Public Health Institute, Addis Ababa P.O. Box 1242, Ethiopia; kalnyou@yahoo.com; 5 Pharmacovigilance and Clinical Trials, Botswana Medicines Regulatory Authority, Gaborone P.O. Box 505155, Botswana; partha18@gmail.com; 6 Center for Innovative Drug Development and Therapeutic Trials for Africa, College of Health Sciences, Addis Ababa University, Addis Ababa P.O. Box 9086, Ethiopia; eyasumakonnen@yahoo.com; 7 Departments of Pharmacology and Clinical Pharmacy, College of Health Sciences, Addis Ababa University, Addis Ababa P.O. Box 9086, Ethiopia

**Keywords:** prevalence, schistosomiasis, STHs, school children, southern Ethiopia

## Abstract

Preventive chemotherapy (PC) is a WHO-recommended strategy to control and eliminate schistosomiasis and soil-transmitted helminths (STHs). We assessed the prevalence, intensity, and correlates of schistosomiasis and STH infection after five rounds of PC in southern Ethiopia. A total of 3162 school children from four schools in Wondo Gennet and Hawella Tula districts were screened for *Schistosoma mansoni* and STHs infection. The overall prevalence of *S. mansoni* infection was 25.8% (range between schools 11.6% to 54.1%), with light (19.1%), moderate (5.3%), and heavy (1.4%) infection intensities. A total of 61.6% *S. mansoni*-infected children were STH co-infected. The overall prevalence of STHs infection was 54.7% (range between schools 30.6–71.0%), with moderate-to-heavy intensity infections being 16.3%. *Ascaris lumbricoides* was the most prevalent 45% (95% CI, 43.5–47) followed by *Trichuris trichiura* 25.3% (95% CI, 23.8–26.9) and hookworm 6.1% (95% CI, 5.3–7). A total of 33.7% of STHs-infected children had *A. lumbricoides* and *T. trichiura* co-infections. *S. mansoni* infection was significantly associated with school and STHs co-infection (*p* < 0.001). STH infection was correlated with school and younger age (*p* < 0.001). Despite repeated PC, *S. mansoni* and STH infection remain significant health problems, and the WHO target to control schistosomiasis and eliminate STH by 2020 may not be achieved. Intensified control and prevention measures, including drug efficacy surveillance, is recommended.

## 1. Introduction

Neglected tropical diseases (NTDs) such as schistosomiasis (also known as bilharziasis) and soil-transmitted helminths (STHs, also known as intestinal worms) remain major public health problems in many parts of the world [1]. Schistosomiasis mainly affects people living in the tropics and sub-tropics, particularly the poor and most deprived communities [2]. Globally, about 252 million people were estimated to be infected with schistosomiasis in 2015 [3], and from these, the sub-Saharan region constitutes more than 90% of the disease burden [2]. Likewise, STHs are the most widespread neglected tropical disease; worldwide more than 1.5 billion people are infected with *Ascaris lumbricoides*, *Trichuris trichiura* (whipworms), and/or hookworm in over 100 endemic countries, together accounting for a major burden of parasitic disease worldwide [4].

Schistosomiasis is endemic in Ethiopia, and intestinal schistosomiasis, caused by *Schistosoma mansoni,* is the most common type of schistosomiasis in the country. About 37.3 million people are currently living in areas where schistosomiasis is endemic in Ethiopia [5]. STHs infections are also widely distributed. Ethiopia is listed among the top five sub-Saharan countries with the highest prevalence of STHs—second, third, and fourth highest for ascariasis, hookworm infection, and trichiurasis, respectively [6]. About 79 million people are currently living in STHs-endemic areas that require preventive chemotherapy [7]. Although all age groups can have schistosomiasis and STHs infections, most infections occur in pre-school and school-aged children.

Schistosomiasis and STHs are more common in rural and disadvantaged urban populations with poor sanitation and lack of a safe water supply. Schistosomiasis is transmitted by contact with infected freshwater [4]. STHs is transmitted by the ingestion of eggs and contact with contaminated water and soil [8]. STHs are transmitted through eggs present in human feces, which contaminate the soil in areas where sanitation is poor. School-aged children are at increased risk of schistosomiasis and STHs infection due to a high level of exposure resulting from contact with water and soil while swimming and playing [9].

Schistosomiasis causes anemia, stunting, fatigue, and diminished physical fitness in children and may also negatively impact cognitive abilities [10]. The severity of morbidity increases as the disease interacts with other parasitic infections like STHs. These parasitic infections are associated with malnutrition, which can negatively impact the quality of life, especially in settings with extreme poverty affecting future growth and development [10,11].

The primary strategy recommended by WHO for the control and elimination of schistosomiasis and STHs infections is through preventive chemotherapy (PC) to all at-risk populations, including school-age children [12]. The large-scale public health intervention involves the administration of praziquantel and albendazole/mebendazole against schistosomiasis and STHs, respectively, to all populations at risk. Annual distribution of praziquantel is recommended in areas where the prevalence is greater than 50%, every two years where the prevalence is between 10−50%, and twice during their primary school age where the prevalence is less than 10% [13].

The WHO and its member states, including Ethiopia, have aimed to control and eliminate schistosomiasis infections as a public health problem by 2020 and 2025, respectively [14]. The NTD roadmap of WHO also aimed, by 2020, to eliminate STHs as a public health problem, defined as <1% of the at-risk population having an infection of moderate or high intensity [4]. Surveillance studies to assess the outcome of long-term program implementation in reducing the disease burden is important to inform policymakers to revise control and elimination strategies to these diseases as public health problems by 2020 and beyond. Since 2014, the national NTD program of Ethiopia has been implementing targeted school-based mass drug administration (MDA) containing praziquantel and albendazole annually in regions with moderate to high prevalence of schistosomiasis and STHs infections [7]. However, no assessment was done on the effectiveness of the implemented interventions. Surveillance data after program implementation to evaluate the impact of long-term PC in reducing the disease burden in endemic countries is critical for evidence-based decision-making. In the current study, we investigated the prevalence, intensity, rates of parasite co-infections, and associated factors of intestinal schistosomiasis and STHs in primary school children in two rural districts of Southern Ethiopia. According to the pre MDA data from the national mapping of NTDs conducted in 2013/2014, both study districts were classified as high prevalence endemic areas (with prevalence of >50%) for both schistosomiasis and STHs [15].

## 2. Results

### 2.1. Socio-Demographic Characteristics

Out of the total of 6184 students enrolled in the four primary schools in the two rural districts, 3162 school children participated in this study. Of these, 56.0% (1772/3162) were from three primary schools in Hawella Tula district, and 44.0% (1390/3162) were from one school in Wondo Gennet district. Hawella Tula district is located along the shore of Lake Hawassa, and Wondo Gennet district is known for its hot water spring. The mean age of the study participants was 11 ± 2 years, and the median was 11 years (IQR = 10–12). Females were 50.3% (1590/3162). Most of the study participants were in the age range of 10–15 years (76.7%) (see Table 1).

### 2.2. Prevalence of Overall Parasite Infection 

Microscopic examinations of stool samples showed that about 64.2% (2030/3162) of the study participants were infected by at least one parasite (either STHs and/or *S. mansoni*). The prevalence of infection varied by study districts. Wondo Gennet district had a higher prevalence (76.1%) compared to Hawella Tula (54.9%). Among the four schools that participated in this study, Wosha primary school in the Wondo Gennet district had the highest prevalence of infection (76.1%), followed by Kidus Pawulos primary school (68.2%), Bushulo primary school (57.2%), and Finchawa primary school (37.1%). The most prevalent parasites were *A. lumbricoids* (45.0%), followed by *S. mansoni* (25.8%), *T. trichiura* (25.2%), and hookworm (6.1%) (refer to Table 2).

The prevalence of infection with any of the parasites significantly varied by age group, being higher in the lower age groups (5–9 years) (68.3%) than in the higher age groups (10–15 years) (63.0%) (*p* = 0.001). The rate of infection also significantly varied by residence and school; Tula and Wosha kebeles had the highest prevalence among the study villages (*p* = 0.001), while Bushulo primary school had the highest among the study schools (*p* = 0.002). The rate of infection significantly varied by kebele (the smallest administrative unit of Ethiopia, similar to ward or neighborhood) where the children live and the school is located. Tula and Wosha kebele had the highest parasite infection rate among the study villages (*p* = 0.001), and Bushulo primary school had the highest parasite infection rate among the study schools (*p* = 0.002). Though not statistically significant, the overall parasite infection rate among males (64.7%) was slightly higher compared to females (63.7%). The details are presented in Table 3.

### 2.3. Prevalence and Intensity of Intestinal Schistosomiasis (S. mansoni)

The overall prevalence of *S. mansoni* infection was 25.8% (815/3162), significantly varying between schools ranging from 11.6% to 54.1% (*p* < 0.001). Among the 25.8% (815/3162) *S. mansoni*-infected children, 74% (603/815) had light infection intensity and 20.7% had moderate. Only 5.3% had heavy infection intensity (detail of intensity by study school is presented in Table 4). The mean egg per gram of stool was 58.1 (95% CI 54.0–62.4). The minimum and maximum egg counts observed per gram of stool were 12 and 1476, respectively. 

In Hawela Tula district, the highest prevalence of *S. mansoni* was observed at Kidus Pawulos primary school (54.1%) followed by Bushulo primary school (20.7%) and Finchawa primary school (11.6%) (Table 4). The prevalence of *S. mansoni* infection was 27.3% at Wosha primary school in Wondo Gennet district. There was no statistically significant association of age, sex, and study districts with *S. mansoni* infection. 

#### Factors Associated with *S. mansoni* Infection

We next assessed factors associated with *S. mansoni* infection, and the correlates were analyzed using univariate followed by multivariate logistic regression (Table 5). Schools were significantly associated with *S. mansoni* infection, being highest in Kidus Pawulos primary school (54.1%) and lowest in Finchawa primary school (11.56%) (*p* < 0.001, see Table 2). Study participants with *T. trichiura* co-infection had significantly higher rates of *S. mansoni* co-infection (35.7%) (286/801) compared to those with no STHs infection (22.4%) (529/2361; *p* < 0.001). S. *mansoni* prevalence was higher among male participants (26.5%) (417/1572) compared to females (25.0%) (198/815), though not statistically significant. 

### 2.4. Prevalence and Intensity of STHs Infection 

The infection rates and intensities of STHs infection were determined for the most prevalent parasites, i.e., *Ascaris lumbricoides*, *Trichuris trichiura,* and hookworm. The overall infection rate of STHs was 54.7% (1731/3162), with significant variations between schools ranging from 30.6% to 71.0% (Table 2). The rates of STHs infection also varied by age group, sex, and area of residence. The most frequently observed helminths were *Ascaris lumbricoides* at 45% (95% CI, 43.5–47), *Trichuris Trichiura* at 25.3% (95% CI, 23.8–26.9), and hookworm at 6.1% (95% CI, 5.3–7). Out of the 1429 children infected with *Ascaris lumbricoides*, 65.2%, 31.7%, and 3.08% had a light, moderate, and heavy infection, respectively. None of the children had a heavy infection with hookworm or *Trichuris trichiura* infections. Moderate intensity of infection for hookworm and *Trichuris trichiura* was also rare. Details of intensity of infection for each parasite are presented in Table 6.

#### Factors Associated with STHs Infection

Factors associated with STHs infection were analyzed using logistic regression (Table 7). Infection with any of the STHs was significantly associated with lower age group, school, and having *S. mansion* co-infection. Rates of STHs infection were significantly different among the four schools, being the highest in the Wosha primary school at 71.0% and the lowest in the Finchawa primary school at 30.7% (*p* < 0.001). The rates of STHs infection were also significantly different between age groups, being higher in the low age group (5–9 years) compared to the 10–15 year old group (*p* < 0.001). No association between sex and STHs infection was found.

### 2.5. Parasite Co-Infections

**Figure 1:**
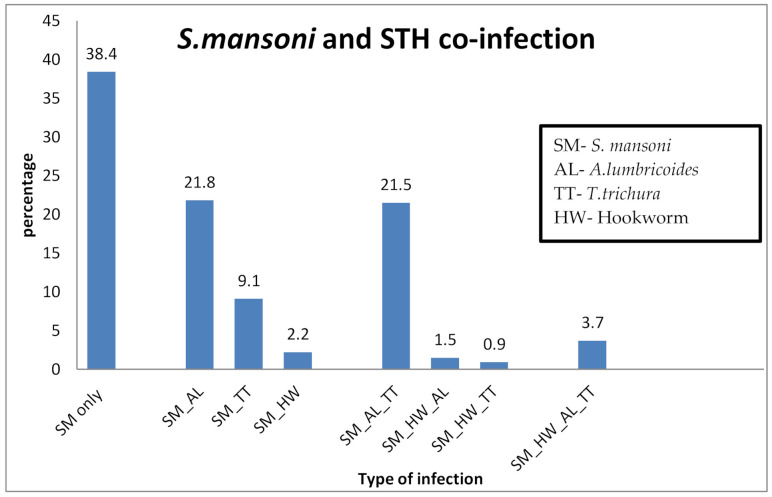
Prevalence of *S. mansoni* infection with or without STHs co-infections.

Our study showed that, among the total *S. mansoni*-infected children, 61.6% (502/815) were co-infected with one or more STHs. Out of the combined *S mansoni* and STHs co-infections, *S. mansoni* with *A. lumbricoides* double infection and *S. mansoni* with *Ascaris lumbricoides* and Trichuris trichiura accounting for 21.8% (178/815) and 21.5% (175/815), respectively, were most prevalent. Details of *S. mansoni* and STHs co-infections are presented in Figure 1.

**Figure 2:**
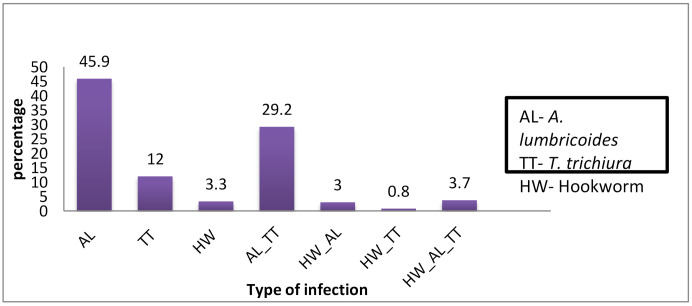
Proportions of single, double, and triple STHs infections.

Out of 1731 students infected with STHs, 62.0% (1073/1731) had a single infection. Among those infected with a single parasite, 73.5% (789/1073) were infected with *A. lumbricoids*. About 33.7% (584/1731) of STHs-infected students had a dual infection. The most prevalent parasites for the double infections were *A. lumbricoides* and *T. trichiura* at 86.6% (506/584). The triple infection rate was 4.3% (74/1731). Only two participants were infected with four parasites. The prevalence and type of co-infection with STHs parasites are presented in Figure 2.

## 3. Discussion

We conducted a school-based cross-sectional study to assess infection prevalence, intensity, and associated risk factors of *S. mansoni* and STHs in two rural districts of southern Ethiopia after implementing five rounds of preventive chemotherapy through the national NTD program. Our main results include the following: i) the overall prevalence of *S. mansoni* infection was 25.8% (ranging between study schools from 11.6% to 54.1%); ii) there was a high overall prevalence of STHs infection (54.7%), with significant variations between schools ranging from 30.6% to 71.0%; iii) a significant correlation was found between *S. mansoni* and STHs, in particular with *T. trichiura*; iii) while school location is a significant predictor of both *S. mansoni* and STHs infection, younger age was an additional significant risk factor for STHs infection. The prevalence of parasite infection significantly varied by the residence area, age, sex, and study schools of participants. To our knowledge, this is the most extensive study to evaluate the status of *S. mansoni* and STH infections after the implementation of multiple rounds of mass drug administration (MDA) in Ethiopia and sub-Saharan Africa at large.

The overall infection rate among children infected at least with one parasite (either STHs or *S. mansoni*) was 64.2% (54.9% at Hawella Tula and 76.1% at Wondo Gennet district). The prevalence of infection with at least one parasite significantly varied between the four study schools, namely 37.1% at Finchawa, 57.2% at Bushulo, 68.2% at Kidus Pawulos, and the highest (76.1%) at Wosha primary schools. The current prevalence of infection with at least one parasite at the Hawella Tula district (54.9%) showed a decreasing trend compared to previous reports of 91.5% in 2007 [16] and 69.7% in 2015 [17]. Program intervention to control infection, including preventive chemotherapy implemented by the Ministry of Health, has resulted in a progressive decline in infection prevalence.

The prevalence of *S. mansoni* infection observed in the two study districts was 25.8%; however, the prevalence significantly varied among the schools, with the highest prevalence observed at Kidus Pawulos School (54.1%) and the lowest observed at Finchawa School (11.6%). District-wise there was no significant variation between Hawella Tula district (24.6%) and Wondo Gennet district (27.3%). Although the prevalence of *S. mansoni* infection varies significantly between the three schools in Hawella Tula district, the overall prevalence rate observed in our study shows a progressive decrease from previous reports in 2007 (73.7%) [16] and in 2015 from the same district (31%) [17]. Similarly, the prevalence of *S. mansoni* infection in the Wondo Gennet district decreased from 74.9% in 2012 [18] and 59.9% in 2014 [19] when compared to the current findings of 27.3% in 2019. This progressive decline in infection prevalence may indicate the impact of control measures, including the implementation of PC in the study areas. However, the current prevalence of 54.1% that was observed at Kidus Pawulos school in this study is much higher than the previously reported 31% in 2015 from the same village. During our data collection, lack of clean water supply for drinking, poor hygiene and sanitation care, and inadequate access to clean toilets were noted. This observation calls for the integration of other environmental control measures such as access to adequate water, sanitation, and hygiene (WASH), and providing health education on basic hygiene and sanitation for behavioral change.

The NTD roadmap set by the WHO targets the control of schistosomiasis, defined as achieving less than 5% of heavy-intensity infection by 2020, and to eliminate schistosomiasis as a public health problem, defined as reaching <1% prevalence of heavy-intensity infections in school-aged children (5–14 years old) by 2025 [14]. The overall heavy-intensity infection (1.4%) for *S. mansoni* infection in this study is near the WHO 2020 target. However, the observed heavy-intensity infection rate at the most affected school (4.6% in Kidus Pawlos) may indicates that the *S. mansoni* infection is still a major public health problem at the study area, and the WHO target to control schistosomiasis by 2020 may be difficult to achieve. Nearly 30% of the infected children from the two most affected schools (Kidus Pawulos and Wosha) had a moderate- or high-intensity prevalence of *S. mansoni* infection (Table 4). This may indicate the presence of hotspots for the continuous transmission of infection in the study area. Hence, additional interventions are urgently needed for the prevention of transmission by treating other high-risk groups, improving hygiene and sanitation, and providing a safe water supply [8] instead of relying on single control measures as are currently implemented by the program.

*S. mansoni* infection was significantly associated with the dwelling area of children and schools. The children from Tula and Wosha kebeles had the highest prevalence of intestinal schistosomiasis infection, and the dwelling area was significantly associated with *S. mansoni* infection. Similarly, children attending Kidus Pawulos primary school also had the highest prevalence of intestinal schistosomiasis, and the school was significantly associated with *S. mansoni* infection. This association is explained by the reason that children living in these areas have high water contact due to the proximity of their dwellings area to Lake Hawassa and local water sources at Wondo Genet. The distribution of *S. mansoni* infection was not gender- or age-associated, which was in agreement with a study conducted in Sanja town [20] and in studies conducted in Madagascar [21] and northwestern Tanzania [22]. This similarity might show that school-aged children, irrespective of their gender and age differences, have equal exposure to contaminated water in their areas.

The overall infection rates of STHs observed in this study was 54.7%, with a significant difference among schools ranging from 30.6% at Finchawa to 71% at Wosha schools. The prevalence also greatly varies among the two study districts, with 42% at Hawella Tula and 71% at Wondo Gennet. The observed STHs infection rates at Hawella Tula district showed a decrease from the previous study reports of 52.4% in 2015. Three soil-transmitted helminths, i.e., *A. lumbricoides*, *T. trichiura*, and hookworm, were predominantly detected. This finding is in agreement with one previous study conducted in one of the study districts (Hawella Tula) in 2015 [17]. *A. lumbricoides* was the most prevalent STHs followed by *T. trichiura* and hookworm infections in both study districts. The high prevalence of STHs observed in the study area might be attributed to the unhygienic use of sugarcane by children, as sugar cane is commonly planted in the study areas. In contrast to the present findings, hookworm infection was found to be predominant in previous studies conducted in different parts of Ethiopia [23,24]. The lower prevalence of hookworm infection observed in the present study might be associated with a decreased less habit of walking barefoot.

The co-infection observed in this study showed 1–4 parasite species were detected per one study participant. Most children harbored double infection with two parasites, and the predominant observed double infections were *A. lumbricoides* with *T. trichiura* with the total co-infection rate exceeding 30%. Over 61.6% of *S. mansoni*-infected children had an infection with at least one STH, perhaps due to similarity in the risk of infection for these parasitic helminths. In children, this co-infection of schistosomiasis and STHs can cause different morbidities like anemia, fatigue, poor growth, and poor cognitive development. These morbidities result in poor school attendance, poor concentration in class, and poor academic performance, which can negatively affect the quality of life of children, impacting their future growth and development [10,11,25,26]. 

The WHO intervention program intends to eliminate STH as a public health problem in children by 2020. WHO defines STHs as a public health problem when more than 1% of the at-risk population has an infection of moderate or high intensity, and its control requires the delivery of one or more public health interventions [4]. Even though the prevalence of STHs infection observed in this study showed a slight decrease from previous reports, the observed high-intensity (1.4%) and moderate-intensity (14.3%) prevalence for *A. lumbricoides* indicates that the national NTD program is far from achieving the target to eliminate STH as a public health problem by 2020. Thus, focusing on the implementation of PC alone may not be adequate, and integration of other control measures, including surveillance of drug efficacy and identifying transmission hotspots for targeted intervention, is urgently needed. Furthermore, since the overall STHs prevalence in the two study districts is >50%, we recommend implantation of biannual MDA for both pre-school and school-age children as well as women of childbearing age as per the recommendation of WHO guideline [27].

## 4. Materials and Methods

### 4.1. Study Area, Population, and Design

**Figure 3:**
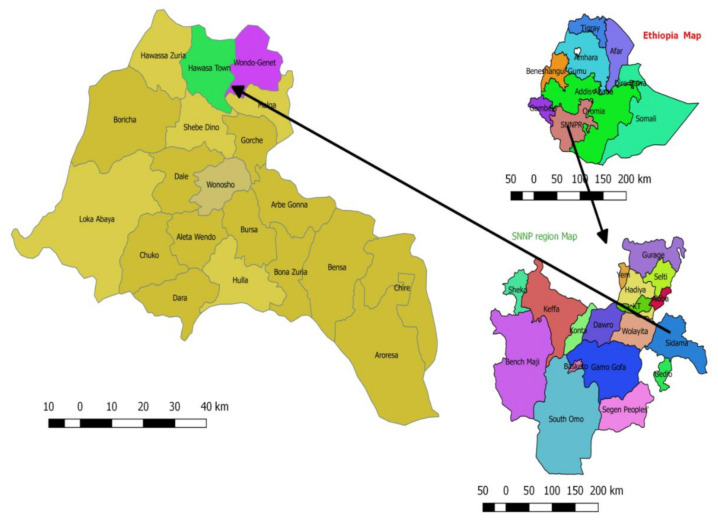
Map of the study site. Top right is the map of Ethiopia located in East Africa. The bottom-right figure shows the map of Southern Nations, Nationalities, and People’s Region where the Sidama Zone is located, where the study districts are found. The figure on the left shows the map of districts participated in the study.

A cross-sectional study was conducted in Hawella Tula (located along the shore of Lake Hawassa) and Wondo Gennet districts located in the Sidama Zone of the Southern Nations, Nationalities, and Peoples’ Region in southern Ethiopia. The two study districts were found to have a high prevalence of STHs and a moderate prevalence of schistosomiasis following a general mapping of NTDs done in Ethiopia between 2013 and 2014. Hawella Tula district is located along the shore of Lake Hawassa at 1699 m above sea level with a temperature range of 7 °C to 38 °C. Residents in Hawella Tula mostly rely on water from Lake Hawassa for their domestic and other uses. Wondo Genet is known for its hot springs, with a warm climate and temperate at an elevation of 1723 m above sea level. The average annual temperature is 17.6 °C with an annual rainfall of 1123 mm. A map of the study districts is presented in Figure 3.

School-going children attending Bushulo, Kidus Pawulos, and Finchawa primary school at Hawella Tula district and Wosha primary school at Wondo Gennet district were included in the study. Schools were selected based on their location near to water source, having a large number of students and classified as high burden of schistosomiasis and STHs as per the 2013/2014 national survey and mapping of NTDS conducted by the Ethiopian ministry of health [28].

### 4.2. Preventive Chemotherapy (PC)

As per WHO recommendations, the frequency of preventive chemotherapy administration depends on infection prevalence rates: i) high-risk areas (infection prevalence rate of ≥50%) to receive mass drug administration (MDA) annually, ii) moderate risk areas (with infection prevalence rates between ≥10 and <50%) to receive MDA at least once every two years, iii) low-risk areas (with infection prevalence of <10%), where MDA should be administered to school children at least twice during their primary education [12]. Based on the national NTD program, PC has been implemented annually since 2014, and the two study districts have received five rounds of school-based mass PC by the end of 2018 (before this study started) [28], and the last PC was in May/2018.

### 4.3. Inclusion and Exclusion Criteria

School children (5–15 years) who reside in the study districts and attend the selected four primary schools were included in the study. Children whose parents/guardians gave informed consent and who provided assent for participation were enrolled.

### 4.4. Data Collection Procedure and Sampling

A structured questionnaire and case record format prepared and piloted for the study was used for collecting socio-demographic and clinical data. Trained schoolteachers and healthcare professionals collected all socio-demographic data for study participants. The sampling of participants was done carefully to ensure balanced age and gender representation in the study.

### 4.5. Detection of Schistosomiasis and STHs

A stool sample was collected from each study participant and immediately transported to the nearby health center using a cold box. Two kato katz smears were prepared from the collected stool sample using a template of 41.7 mg following the standard protocol [29]. The slides were read by two different trained laboratory professionals independently at each health center. Egg counts from both slides were recorded, and the average value was taken and converted to eggs per gram of stool using a factor of 24 [29,30]. The intensity of infection was determined for each parasite as light, moderate, and heavy based on WHO criteria [31,32], specified as follows:

- *S. mansoni* (light (1–99 epg), moderate (100–399 epg), heavy (≥400 epg));
- *T. trichiura* (light (1–999 epg), moderate (1000–9999 epg), heavy (≥10,000 epg));
- *lumbricoides* (light (1–4999 epg), moderate (5000–49,999 epg), heavy (≥50,000 epg));
- Hook worm (light (1–1999 epg), moderate (2000–3999 epg), heavy (≥4000 epg)).

Trained laboratory professionals from nearby health centers and Hawassa university hospital read slides, and internal and external quality control was applied using senior experienced laboratory professionals from the Ethiopian Public Health Institute.

### 4.6. Statistical Analysis

Data entry and cleaning were done using Excel and transported to Stata version 11 for analysis. Descriptive statistical analysis was used to determine the prevalence of *S. mansoni* and STHs. Factors associated with the infections were determined using univariate and multivariate logistic regression analysis. Univariate followed by multivariate analysis was done to determine factors associated with infection. Variables with *p*-value < 0.2 from the univariate analysis were included in a multivariate analysis using a backward/forward selection method to fit the model. Probability values of less than or equal to 0.05 (*p* < 0.05) were considered statistically significant.

### 4.7. Ethical Considerations

The study obtained ethical approval from the Southern Nations, Nationalities, and People’s Region Health Bureau ethical clearance committee with a reference number of 9026-19/14966. Before initiating the study, an awareness creation meeting was held with regional health and education bureaus, zonal and woreda health and education offices, and school and health center heads, schoolteachers, school administrators, and students. During the meeting, the objectives of the study, the study procedures, and samples to be taken were explained. Participation was fully voluntary, and informed verbal consent from parents/guardians and verbal assent from children 12 years and above was obtained.

## 5. Conclusions

After five rounds of PC, *S. mansoni* and STHs infection were highly prevalent in the study area, with 64.2% of the children having at least with one parasite (either STHs or *S. mansoni*). The infection rates varied significantly between the study schools, ranging from 11.6% to 54.1% for *S. mansoni* and 30.6% to 71.0% for STHs. The finding of *S. mansoni* infection in >50% and STHs infection in >70% of the children attending the most highly affected school indicates hotspots for community transmissions. Based on our findings, the WHO target to control schistosomiasis and eliminate STHs as a public health problem by 2020, respectively, may not be achieved in the current setting. Our findings provide valuable information for national NTD programs and policymakers to revise intervention strategies to control and eliminate schistosomiasis and STHs infection as public health problems. Besides preventive chemotherapy, a surveillance system to assess anthelmintic drug efficacies for early detection of parasite resistance and other integrated control measures such as the provision of safe water supply, improving sanitation, access to clean toilets, and snail control programs need to be implemented.

## Figures and Tables

**Table 1:** Socio-demographic characteristics of study participants.

Characteristics	Category	*n*	%
Age group in years	5–9	737	23.3
	10–15	2425	76.7
Sex	Female	1590	49.7
	Male	1572	50.3
Schools	Bushulo	1095	34.6
	Kidus Pawulos	372	11.8
	Finchawa	305	9.7
	Wosha	1390	43.9
Kebele	Tula	1400	44.2
	Finchawa	372	11.8
	Wosha	1390	44
District	Hawella Tula	1772	56
	Wondo Gennet	1390	44
Total		3162	100

**Table 2:** Prevalence of any parasite infection stratified by socio-demographic characteristics, school, and district of residence.

Characteristics		Screened Participants	Infection Prevalence (STHs and/or *S. mansoni)*	*S. mansoni*-Infected	STHs-Infected
		*n* (%)	*n* (%)	*n* (%)	*n* (%)
Total		3162	2030 (64.2%)	815 (25.8%)	1731 (54.7%)
Age group in years	5–9	737 (23.3%)	503 (68.3%)	189 (25.6%)	448 (60.8%)
	10–15	2425 (76.7%)	1527(63.0%)	626 (25.8%)	1283 (52.9%)
Sex	Female	1590 (49.7%)	1013 (63.7%)	398 (25.0%)	862 (54.2%)
	Male	1572 (50.3%)	1017 (64.7%)	417 (26.5%)	869 (55.3%)
School	Bushulo	1095 (34.6%)	626 (57.2%)	227 (20.7%)	507 (46.3%)
	Kidus Pawulos	305 (9.7%)	208 (68.2%)	165 (54.1%)	123 (40.3%)
	Finchawa	372 (11.8%)	138 (37.1%)	43 (11.6%)	114 (30.6%)
	Wosha	1390 (43.9%)	1058 (76.1%)	380 (27.3%)	987 (71.0%)
District	Hawella Tula	1772 (56.0%)	972 (54.9%)	435 (24.6%)	744 (42.0%)
	Wondo Gennet	1390 (44.0%)	1058 (76.1%)	380 (27.3%)	987 (71.0%)

**Table 3:** Rates and correlates of parasite infection (either soil-transmitted helminth (STH) and/or *S*. *mansonai)* using logistic regression.

Characteristics	Category	Prevalence of Any Parasite Infection		Univariate			Multivariate		
		*n*	%	COR	95% CI	*p*-Value	AOR	95% CI	*p*-Value
Age group in years	5–9	503/737	68.3	1.3	1.1–1.5	0.009	1.4	1.1–1.6	0.001
	10–15	1527/2425	63.0	1 ^a^					
Sex	Female	1013/1590	63.7	1 ^a^					
	Male	1017/1572	64.7	1.04	0.09–1.21	0.564			
School	Bushulo	626/1095	57.2	2.3	1.8–2.9	0.000	2.4	1.8–3.0	0.000
	Kidus Pawulos	208/305	68.2	3.7	2.6–5.0	0.000	3.7	2.6–5.0	0.000
	Finchawa	138/372	37.1	1 ^a^					
	Wosha	1058/1390	76.1	5.4	4.2–6.9	0.000	5.6	4.4–7.1	0.000

In the adjusted model, we adjusted for age and school. Due to co-linearity, village/kebele and district were not included in the model. ^a^ = reference, COR = crude odds ratio, AOR = adjusted odds ratio, CI = confidence interval.

**Table 4:** Prevalence and intensity of *S. mansoni* infection by schools.

School	*S. mansoni* Infection Prevalence	Intensity of Infection			
		No Infection	Light	Moderate	Heavy
Overall intensity	25.8%	74.2%	19.1%	5.3%	1.4%
Bushulo	20.7%	79.3%	18.4%	2.3%	0.01%
Kidus Pawulos	54.1%	45.9%	33.8%	15.7%	4.6%
Finchawa	11.6%	88.4%	11%	0.05%	0
Wosha	27.3%	72.7%	18.6%	6.8%	2%

**Table 5:** Prevalence and correlates of *S. mansoni* infection among school-going children.

Characteristics	Category	*S. mansoni* Infection	Univariate			Multivariate		
		n %	COR	95% CI	*p*-Value	AOR	95% CI	*p*-Value
Age group in years	5–9	189 (25.6)	1 ^a^					
	10–15	626 (25.8)	1.0	0.8–1.2	0.337			
Sex	Female	398 (25.0)	1 ^a^					
	Male	417 (26.5)	1.1	0.9–1.3	0.926			
School	Bushulo	227 (20.7)	2.0	1.4–2.8	0.000	1.8	1.3–2.6	0.001
	Kidus Pawulos	165 (54.1)	9.0	6.1–13.3	0.000	8.7	5.9–12.8	0.000
	Wosha	380 (27.3)	2.9	2.1–4.0	0.000	2.3	1.6–3.2	0.000
	Finchawa	43 (11.6)	1 ^a^					
Hookworm	Yes	67 (34.5)	1.6	1.2–2.1	0.004	1.3	0.9–1.8	0.07
	No	748 (25.2)	1 ^a^					
*A. lumbricoides*	Yes	399 (27.9)	1.2	1.1–1.4	0.012	1	0.8–1.2	0.87
	No	416 (24.0)	1 ^a^					
*Trichuris trichiura*	Yes	286 (35.7)	1.9	1.6–2.3	0.000	1.9	1.6–2.3	0.000
	No	529 (22.4)	1 ^a^					

Data were analyzed using logistic regression. In the adjusted model, we adjusted for school, hookworm, *A. lumbricoides*, and *T. trichiura.* Due to co-linearity, village/kebele and district were not included in the model. ^a^ = reference, COR = crude odds ratio, AOR = adjusted odds ratio, CI = confidence interval.

**Table 6:** Prevalence of STH infections stratified by infection intensities for each parasite.

Parasite	Overall Infection Prevalence	Intensity of Infection			
		No Infection	Light	Moderate	Heavy
*A. lumbricoides*	45%	54.8%	29.5%	14.3%	1.4%
*T. trichiura*	25.3%	74.7%	25%	0.3%	0
Hookworm	6.1%	93.8%	6.0%	0.03%	0

**Table 7:** Rates and correlates of STH infections among school-going children.

Characteristics	Category	STHs Pos		Univariate			Multivariate		
		*n*	%	COR	95% CI	*p*-Value	AOR	95% CI	*p*-Value
Age group in years	5–9	448	60.8	1.4	1.2–1.6	0.000	1.6	1.3–1.9	0.000
	10–15	1283	52.9	1 ^a^					
Sex	Female	862	54.2	1 ^a^					
	Male	869	55.3	1.0	0.9–1.2	0.547			
School	Bushulo	507	46.3	1.9	1.5–2.5	0.000	2	1.6–2.6	0.000
	Kidus Pawulos	123	40.3	1.5	1.1–2.1	0.009	1.3	0.9–1.8	0.12
	Wosha	987	71.0	5.5	4.3–7.1	0.000	5.6	4.3–7.2	0.000
	Finchawa	114	30.7	1 ^a^					
*S. mansoni*	Yes	502	29.0	1.5	1.2–1.7	0.000	1.5	1.2–1.8	0.001
	No	313	21.9	1 ^a^					

Data were analyzed using logistic regression. In the adjusted model, we adjusted for age, school, and *S. mansoni* infection. Due to co-linearity, village/kebele and district were not included in the model. ^a^ = reference, COR = crude odds ratio, AOR = adjusted odds ratio, CI = confidence interval.
